# Wider recognition and greater understanding of postinfectious, antibiotic-refractory Lyme arthritis

**DOI:** 10.1172/JCI184109

**Published:** 2024-09-03

**Authors:** Allen C. Steere, Jacob E. Lemieux

**Affiliations:** 1 Center for Immunology and Inflammatory Diseases, Division of Rheumatology, Allergy, and Immunology and; 2 Division of Infectious Diseases, Massachusetts General Hospital, Harvard Medical School, Boston, Massachusetts, USA.

## Abstract

Lyme disease, caused by *Borrelia burgdorferi* (*Bb*), can progress to Lyme arthritis (LA). While most patients with LA respond successfully to antibiotic therapy, a small percentage fail to improve, a condition known as antibiotic-refractory Lyme arthritis (ARLA). While T cell responses are known to drive ARLA, molecular mechanisms for ARLA remain unknown. In this issue of the *JCI*, Dirks et al. isolated disease-specific Th cells from patients with ARLA residing in Germany. A distinct TCR-β motif distinguished ARLA from other rheumatic diseases. Notably, the TCR-β motif was linked predominantly to HLA-DRB1*11 or 13 alleles, which differed from alleles in patients from North America. It also mapped primarily to T peripheral helper (Tph) cells, as opposed to classical Th1 cells. These findings provide a roadmap explaining how T cell responses necessary for control of an infection can, despite antibiotic therapy, drive a disadvantageous T cell response, resulting in a postinfectious, inflammatory arthritis.

## Wider Recognition of ARLA as a postinfectious complication

In 1976, Lyme arthritis (LA) was recognized as a separate clinical entity because of geographic clustering of children in the Lyme Connecticut area who had often been thought to have juvenile rheumatoid arthritis (1). With systematic study, it became apparent that the natural history of LA was quite variable, ranging from brief episodes of arthralgia to several attacks of intermittent swelling and pain in one or both knees, to persistent (chronic) arthritis, usually in one knee, sometimes associated with erosion of cartilage and bone (2).

After identification of the causative agent, *Borrelia burgdorferi* (*Bb*) in 1982, antibiotic treatment studies showed that most LA patients could be treated successfully with one- or two-month courses of oral antibiotic therapy, and, if necessary, with intravenous (IV) antibiotics (3). However, a small percentage of patients had minimal, if any, improvement with oral and IV antibiotics, which was called postantibiotic or antibiotic-refractory Lyme arthritis (ARLA). In these patients, culture and PCR testing for *Bb* in synovial tissue were uniformly negative in the postinfectious period (4), and, after antibiotic therapy, the patients were treated successfully with disease-modifying antirheumatic drugs (DMARDs), such as methotrexate and TNF inhibitors, suggesting that ARLA is a postinfectious complication (3).

Although the original report of LA in Germany described several patients with persistent (chronic) LA (5), subsequent reports concluded that joint involvement in European LA was generally less common than in the U.S., it usually occurred earlier in the illness, and it was less inflammatory (6). However, using a National Database, 37 patients with LA from France were recently reported, based on positive PCR and serology results in joint fluid, including 3 patients with ARLA (7). After antibiotic therapy, these three patients developed oligoarticular or polyarticular arthritis, which was treated successfully with DMARD therapy. In the U.S., other systemic autoimmune diseases, including rheumatoid arthritis (RA), peripheral spondyloarthritis, or psoriatic arthritis, have been noted within months after Lyme disease (8). Although no other type of arthritis could be identified in the three French patients, mechanistic information was not available to help make the distinction between ARLA or possible infection-induced triggering of another form of chronic inflammatory arthritis.

## Immunopathogenesis of ARLA

In this issue of the *JCI*, Dirks et al. showed definitively by elucidation of a unique molecular program of disease-specific Th cells that ARLA occurring in diverse regions of Germany had similar features as ARLA cases in North America (9). Despite antibiotic therapy, all 13 patients, who were teenagers, presented with antibiotic-refractory, chronic arthritis and received an intraarticular steroid injection. All but one experienced subsequent arthritis and required treatment with DMARDs. However, in contrast with ARLA risk alleles (HLA-DRB1*04, 0101, or 1501) in U.S. patients (10), 5 of the 13 German patients had the DRB1*1101 allele, which appeared to be protective in U.S. patients.

In North America, the basic pathologic feature of ARLA is the development of an excessive, dysregulated proinflammatory immune response during infection of joints with highly inflammatory *Bb* strains (11). This response is characterized by high IFN-γ levels and low levels of the antiinflammatory cytokine IL-10, which persists and may even worsen in the postinfectious period (11). The consequences of this response in synovia of patients with ARLA include vascular damage, autoimmune and cytotoxic responses, and massive fibroblast proliferation and fibrosis, a lesion similar with that found in other forms of chronic inflammatory arthritis, including in RA or juvenile idiopathic arthritis (JIA).

## Characteristics of pathogenic T helper cells in ARLA

In the Dirks work (9), the phenotype of these T effector cells was further defined. Using PD-1 and HLA-DR expression to identify recently antigen-activated T cells, they found a high frequency of oligoclonally expanded PD-1^hi^ HLA-DR^+^CD4^+^ T cells in synovial fluid (SF) but not in peripheral blood of the patients. Despite antibiotics and subsequent DMARD therapy, this cell type persisted in affected joints for up to 2.5 years after the onset of arthritis. Moreover, a comparable frequency of this cell type was found in both SF and synovial tissue. Using single RNA-Seq spatial microscopy, they showed that PD-1^hi^ HLA-DR^+^CD4^+^ T cells localized to the center of lymphoid aggregates a short distance from CD20^+^ B cells, suggestive of ongoing T/B cell interactions.

TCR sequencing of PD-1^hi^ HLA-DR^+^CD4^+^ T cells revealed TCR clusters that were predicted to bind the same MHC-restricted peptide antigen (9). The combination of a CDR3-β motif and a CDR1-β motif had the highest sensitivity and specificity for ARLA, which the authors named the “ARLA TCR-β motif”. The CDR3-β sequences of the most frequent specificity groups in the cluster had greater nucleotide than amino acid diversity, which is a characteristic sign of a T cell response converging toward a specific set of antigens.

## Distinguishing ARLA from other rheumatic diseases

The ARLA TCR-β motif was also valuable in distinguishing ARLA from other rheumatic diseases (9). A cluster of T cells with this motif was found in 93.6% of patients with ARLA, all of whom had the HLA-DRB1*11 or DRB1*13 allele. This motif was not found in JIA or RA patients, ruling out the possibility of *Bb*-induced triggering of these other rheumatic diseases. The HLA-DRB1*11 and DRB1*13 alleles could be distinguished from other HLA-DRB1*alleles in their patient cohort by the presence of a serine at position 13 (P13) of the HLA-DR molecule, a position that influences peptide binding. HLA-DRB1 position 13 has a strong association with CDR3-β, which mediates risk for multiple autoimmune diseases, including JIA (12).

## Function of T cells with the ARLA motif

Rather than classical Th1 cells, this ARLA TCR-β motif mapped primarily to T peripheral helper (Tph) cells, which displayed signs of sustained proliferation, continuous TCR signaling, and expression of CXCL13, a B cell chemoattractant, and of IFN-γ, a major proinflammatory cytokine (9). Tph cells drive disease pathogenesis in several autoimmune diseases, including RA and JIA (13, 14). Similarly, in previous studies, patients with ARLA in the U.S. had exceptionally high levels of CXCL9 and CXCL10 in joint fluid (15), which are IFN-γ–dependent chemoattractants for CD4^+^ and CD8^+^ T cells; more than half of upregulated genes in their synovial tissue were IFN-response genes (11). In coculture experiments, their Teff cells secreted large amounts of IFN-γ and TNF-α, but the small number of Tregs, which secreted only small amounts of IL-10, were inadequate for suppression of Teff cells (16). This proinflammatory immune response is heightened and the antiinflammatory response is dampened in patients with LA who have one nucleotide substitution (1805GG rather than 1805GT or TT) in the gene encoding the TLR-1, which is a risk factor for ARLA (15).

## Autoimmune targets in ARLA

Autoimmune diseases often have strong associations with specific HLA-DR alleles, as is the case with ARLA in North America and Europe. However, in European LA, the *Borrelia* antigens or self antigens recognized by these cells are not yet known. In patients with ARLA in the U.S., recognition of an HLA-DRB1*0401–restricted epitope of *Bb* outer-surface protein A (OspA^163-175^) has been associated with more frequent ARLA (10), which leads to a strong Th1 response thereby helping to set the stage for ARLA. Additionally, by elution of HLA-DR–presented peptides directly from ARLA synovial tissue, T cell epitopes of three vascular-associated and four extracellular matrix–associated autoantigens were identified and shown to be targets of T and B cell responses that are associated with synovial pathology (11, 17). Patients with T cell responses to ECM epitopes had increased frequencies of DRB1*04 or 1501 alleles compared with patients with antibiotic-responsive arthritis (54% versus 5%, *P* = 0.0008) (17). Variants in the gene locus encoding late cornified epithelial protein (LCE3) correlate with increased levels of Th17 mediators and with autoantibody responses to vascular self antigens (18).

In addition to the greater understanding of the molecular program of T cells afforded by the Dirks study (9), it will be important to gain greater understanding of the role of B cells in ARLA pathogenesis. Compared with patients with antibiotic-responsive LA, those with ARLA have greater antibody-dependent complement deposition (AFCD) (19), and autoantibody-dependent cell-mediated cytotoxicity appears to be involved in the pathogenesis of obliterative microvascular lesions in synovia in ARLA (20).

## Strain variation in *Bb* in North America and Europe

Because genetic backgrounds of patients in New England in the U.S. and patients in Germany are likely similar, we speculate that differences in *Bb* strain and in the T cell epitopes recognized are the likely explanation for the different HLA-DR alleles associated with ARLA in Germany and North America. Four species of Lyme disease *Borrelia* cause Lyme disease in Europe, and *Bb* sensu stricto (*Bbss*) is the least common species (fewer than 10% of cases), whereas in the US, *Bbss* causes almost all cases of Lyme disease (21). In the recent French study (7), *Bbss* was the most common cause of LA (54% of patients), suggestive of skewing toward *Bbss* as the most common cause of arthritis there. Moreover, in North America, the *Bbss* strain specified by OspC type A or according to another typing system, by ribosomal spacer type 1 (RST 1) is prominent in New England, and infection with this strain induces greater inflammation, more severe disease, and more frequent ARLA than other U.S. or European strains tested (15, 22).

Only limited information is available on the genetic structure of *Bb*ss from Europe, but the available genomes show striking differences between populations in the U.S. and Europe. In a recent study, 299 *Bbss* isolates from the northeastern U.S. and from Slovenia underwent whole gene sequencing (WGS) (23). In the U.S., WGS A isolates, which includes OspC type A, were prominent. These isolates possessed the largest pangenome, encoded the greatest number of sequences for outer-surface lipoproteins, and disseminated at the highest rates in human disease. In contrast, European *Bb* WGS A isolates had sequences for fewer lipoproteins. It will be important to learn the characteristics of *Bb* strains that lead to ARLA in Europe, which may provide additional clues to microbial components that trigger ARLA.

## Conclusions and implications

In addition to wider geographic recognition of ARLA, the Dirks study confirms and extends the current paradigm that, despite appropriate antibiotic therapy, ARLA is a postinfectious complication requiring DMARD therapy. Importantly, their study provides a roadmap for better understanding how a T cell response necessary for control of an infection transitions and drives a disadvantageous T cell response leading to postinfectious arthritis. In addition to the help this knowledge may provide in more accurate diagnosis and treatment of ARLA, this roadmap has broad implications for better understanding the interactions of infection and autoimmunity in other rheumatic diseases (24). For example, in RA, identification of triggering commensal microbes and features of the immune response necessary for arthritogenicity promise a new age in understanding this most common form of autoimmune, chronic inflammatory arthritis, which will likely create new paradigms for improving both diagnosis and treatment.

## Version 1 09/03/2024

Electronic publication
